# Distinct metabolic responses of an ovarian cancer stem cell line

**DOI:** 10.1186/s12918-014-0134-y

**Published:** 2014-12-18

**Authors:** Kathleen A Vermeersch, Lijuan Wang, John F McDonald, Mark P Styczynski

**Affiliations:** School of Chemical & Biomolecular Engineering and Parker H. Petit Institute for Bioengineering & Bioscience, Georgia Institute of Technology, 311 Ferst Dr, Atlanta, GA 30332-0100 USA; Ovarian Cancer Institute, School of Biology, and Parker H. Petit Institute of Bioengineering and Bioscience, Georgia Institute of Technology, 315 Ferst Dr, Atlanta, GA 30332-0363 USA

**Keywords:** Metabolomics, Cancer stem cells, Ovarian cancer, Cancer metabolism, Biologically-inspired metabolic perturbations

## Abstract

**Background:**

Cancer metabolism is emerging as an important focus area in cancer research. However, the *in vitro* cell culture conditions under which much cellular metabolism research is performed differ drastically from *in vivo* tumor conditions, which are characterized by variations in the levels of oxygen, nutrients like glucose, and other molecules like chemotherapeutics. Moreover, it is important to know how the diverse cell types in a tumor, including cancer stem cells that are believed to be a major cause of cancer recurrence, respond to these variations. Here, *in vitro* environmental perturbations designed to mimic different aspects of the *in vivo* environment were used to characterize how an ovarian cancer cell line and its derived, isogenic cancer stem cells metabolically respond to environmental cues.

**Results:**

Mass spectrometry was used to profile metabolite levels in response to *in vitro* environmental perturbations. Docetaxel, the chemotherapeutic used for this experiment, caused significant metabolic changes in amino acid and carbohydrate metabolism in ovarian cancer cells, but had virtually no metabolic effect on isogenic ovarian cancer stem cells. Glucose deprivation, hypoxia, and the combination thereof altered ovarian cancer cell and cancer stem cell metabolism to varying extents for the two cell types. Hypoxia had a much larger effect on ovarian cancer cell metabolism, while glucose deprivation had a greater effect on ovarian cancer stem cell metabolism. Core metabolites and pathways affected by these perturbations were identified, along with pathways that were unique to cell types or perturbations.

**Conclusions:**

The metabolic responses of an ovarian cancer cell line and its derived isogenic cancer stem cells differ greatly under most conditions, suggesting that these two cell types may behave quite differently in an *in vivo* tumor microenvironment. While cancer metabolism and cancer stem cells are each promising potential therapeutic targets, such varied behaviors *in vivo* would need to be considered in the design and early testing of such treatments.

**Electronic supplementary material:**

The online version of this article (doi:10.1186/s12918-014-0134-y) contains supplementary material, which is available to authorized users.

## Background

Since 1924, when Warburg discovered aerobic glycolysis, it has been known that cancer cellular metabolism is distinct from normal cellular metabolism [1,2]. It is only recently that the important role metabolism plays in cancer has become more generally recognized. Dysfunctional metabolism is now acknowledged as a hallmark of cancer, and multiple different examples of altered metabolism in cancer cells have been demonstrated [3,4]. Most cancer cellular metabolism studies are usually performed using *in vitro* cell culture. Cell culture conditions are ideal: an overabundance of an energy source (usually in the form of glucose) is supplied, oxygen concentration is kept high, and cells are grown in monolayers to keep nutrient and oxygen transfer high to all cells. Unfortunately, these *in vitro* conditions drastically differ from the conditions found *in vivo* in the tumor environment, which are far from ideal. With the fast growth of tumors, angiogenesis cannot occur quickly enough to supply the entire tumor with capillaries, resulting in nutrient fluctuations, hypoxia, and ischemia (decreased blood supply to the tumor causing a state of depleted oxygen and nutrients) – particularly in the center of tumor. Along with poor cellular growth conditions, most tumors are also treated with chemotherapeutics to attempt to eradicate the tumor. These differences in environmental conditions can actually be critical in correctly understanding and treating cancer cells. For example, differences between *in vitro* cellular growth conditions and the *in vivo* tumor environment have been identified as responsible for the inconsistency in clinical and *in vitro* lethal concentrations for metformin [5]. It is thus critical to attempt to study cancer cells grown *in vitro* under conditions relevant to their natural tumor environment.

Another important characteristic of tumors that may not be well-represented in *in vitro* models is the heterogeneous population of cancer cells. Part of the heterogeneous population are cells referred to as cancer stem cells due to their stem-like properties: they can differentiate and self-renew, and they are chemo- and radio-resistant [6]. Cancer stem cells are thought to be a primary cause of cancer recurrence; it is thus critical to characterize and understand the behavior of cancer stem cells, as failure to eradicate these cells in addition to bulk tumor cells may contribute to the high mortality rates of some types of cancer, including ovarian cancer [6-8].

Thus, to fully model tumor metabolism, we must characterize the metabolism of both established cancer cell and cancer stem cell lines, and we must do this in contexts with as much relevance to the *in vivo* tumor environment as possible. Differences in metabolic behaviors between these two cell types could allow us to start to understand how the different cell types handle some of the stresses encountered in a tumor. Understanding the metabolic effects of these stresses could lead to a more complete model of cancer pathology and the development of metabolism-targeted or cancer stem cell-targeted therapies.

Here, we use a recently-established model system consisting of OVCAR-3 ovarian cancer cells (OCCs) and the isogenic ovarian cancer stem cells (OCSCs) derived directly from the OCCs to perform the first-ever *in vitro* characterization of the metabolic responses of these cell types to environmental conditions related to the *in vivo* environment [9]. Ovarian cancer is a particularly relevant model system here because it has such a high rate of recurrence, making the study and understanding of ovarian cancer stem cells potentially quite important. We subjected both cell types to phsyiologically inspired environmental *in vitro* perturbations and measured their metabolic responses using mass spectrometry. Since OCCs and OCSCs are already known to exhibit significant metabolic differences during normal growth [10], we hypothesized that these two cell types may also have distinct metabolic responses to environmental perturbations associated with the *in vivo* tumor environment. The perturbations used in this study were glucose deprivation, hypoxia, glucose deprivation combined with hypoxia (to model ischemia), and chemotherapeutic treatment. Docetaxel, a common first line treatment for ovarian cancer, was chosen as the chemotherapeutic. These perturbations were applied over a period of 48 hours with metabolomics measurements being made throughout that period using two-dimensional gas chromatography–mass spectrometry (GCxGC-MS).

## Results and discussion

### Metabolic analysis discovers numerous metabolic changes for OCCs upon docetaxel treatment

To profile cellular metabolism, GCxGC-MS was used to analyze the intracellular samples collected during docetaxel treatment for OCCs. Both unknown analytes and annotated analytes result from this data processing pipeline. Because of the limited scope of metabolite mass spectrum databases and the conservative identification cutoff we employ in data processing, some of the unannotated analytes may be endogenous metabolites that have not yet had a mass spectrum deposited in a database, or their mass spectral similarities to library spectra may fall under our conservative naming cutoff. Therefore, these unknown analytes can still be important to the metabolic profile of the cells, and so they were included for most downstream analyses except enrichment analyses (which require metabolite identities). Lists of annotated metabolites in this work come from unique matches to known human metabolites in either the Kyoto Encyclopedia of Genes and Genomes (KEGG) or the Human Metabolome Database (HMDB), followed by a manual confirmation of similarity between the annotated peak spectrum and the library spectrum. For the OCCs, 177 reproducible analytes were detected overall and 44 unique metabolites were mapped to either KEGG or HMDB. Metabolite levels for the entire data set are shown in Additional file 1: Figure S3.

Two-way ANOVA was used to identify FDR-corrected p values for group, time, and interaction effects. Group effects relate to the difference in analytes between the experiment conditions, the time effect measures how the analyte changes with time across all the conditions, and the interaction effect captures effects where the combination of time and group effects are not additive. Two-way ANOVA on OCC intracellular data revealed many more analytes that were significantly different between the two docetaxel treatments (IC_50_ and 1.5× IC_50_) and control cells than were found using traditional one-way ANOVA. The numbers of metabolites with statistically significant effects for both one-way and two-way ANOVA are shown in Additional file 2: Table S1 and Table S2. The annotated metabolites identified as having statistically significant group effects are shown in Table 1 for both docetaxel concentrations and for each concentration individually. All of the metabolites identified as statistically significant for both docetaxel concentrations were also found as statistically significant via *t* tests for at least one of the concentrations individually.

**Table 1:** **Metabolites identified as individually statistically significantly different during chemotherapeutic treatment using two-way ANOVA for OCCs and OCSCs**

		**Metabolite**	**Group**	**Time**	**Interactions**
OCC	Control vs IC_50_ vs 1.5 × IC_50_	2-Butyl-1-octanol	6.88E-04	1.48E-07	1.96E-07
		Hexadecane	2.83E-03	4.81E-11	0.831
		D-Glucose	2.83E-03	7.21E-03	1.73E-03
		Uracil	4.50E-03	1.05E-11	1.96E-07
		L-Tyrosine	6.00E-03	6.35E-08	2.26E-03
		Erythronic acid	7.07E-03	6.16E-03	0.424
		Xylobiose	0.047	0.140	0.162
	Control vs IC_50_	2-Butyl-1-octanol	0.016	3.03E-06	4.81E-05
		Uracil	0.039	4.28E-07	1.53E-04
		D-Glucose	0.039	1.07E-03	0.011
		Hexadecane	0.041	1.93E-07	0.766
		L-Tyrosine	0.041	2.01E-06	1.19E-03
		Erythronic acid	0.041	0.089	0.644
		Erythritol	0.041	0.281	0.484
	Control vs 1.5 × IC_50_	Uracil	0.025	2.84E-08	3.09E-06
		Hexadecane	0.025	3.41E-07	0.510
		L-Tyrosine	0.025	1.05E-04	0.013
		D-Glucose	0.025	0.041	7.10E-03
		Erythronic acid	0.025	0.098	0.496
OCSC		Ethanolamine	4.82E-03	2.76E-05	1.39E-05

**Figure 1:**
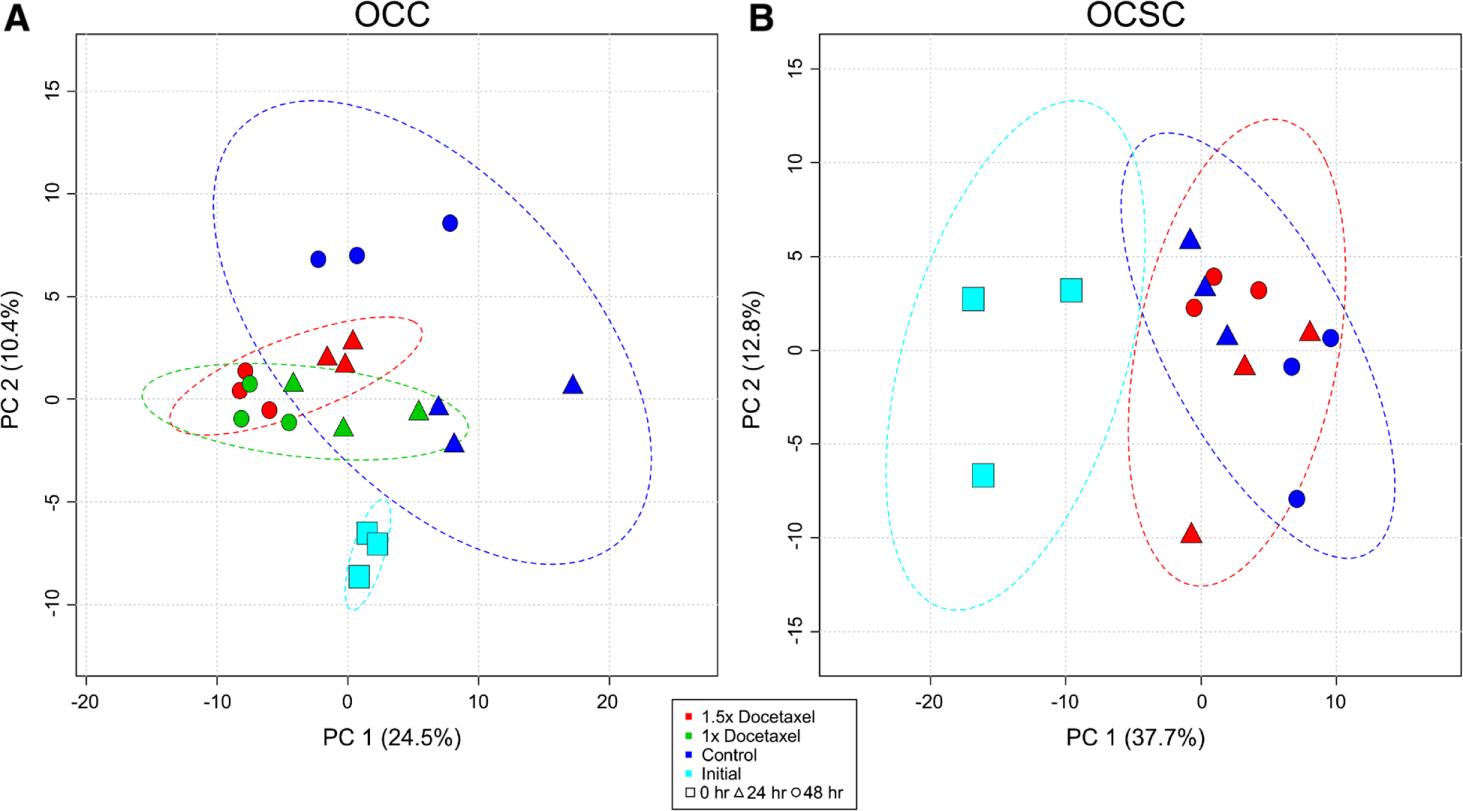
**PCA of docetaxel perturbations highlights the different metabolic responses of OCCs and OCSCs.** PCA of metabolic profiles of OCCs **(A)** and OCSCs **(B)** in response to docetaxel over 48 hours. Dotted ovals represent 95% confidence intervals of the membership of each sample class. **(A)** PCA shows clear separation between control and docetaxel treated OCCs, as well as separation between the two treatment levels, and separation between the time points for all treatments and controls. PC1 and PC2 both are responsible for the separation between different experimental groups as well as time. **(B)** PC1 separates the initial time point and the later time points, but there is no separation between control and docetaxel treated cells, suggesting that docetaxel has little to no effect on the metabolism of OCSCs over a 48 hour period.

Principal component analysis (PCA) allows for graphical interpretation of data through unsupervised dimensional reduction. The clear effect of docetaxel on OCC metabolism is further supported with PCA, as seen in Figure 1A. Docetaxel-treated samples cluster together away from both the control and initial samples with distinct separation between the different concentrations and time points. Even though the separation is small between the different time points and between the different treatment levels (each in different PCs), the differences are clear and consistent. The control samples cluster together high in PC1 or PC2, with large variation between the 24 hour samples (which lie closer to the initial samples) and the 48 hour samples. The control OCCs have a much larger variance between the 24 and 48 hour time points compared to the variance between docetaxel treated OCCs. PC1 and 2 seem to be equally responsible for capturing the separation between treatment and time points.

Metabolic pathway enrichment analysis (MPEA) in MetaboAnalyst identified 15 pathways that were significantly enriched for differences between control and docetaxel treated OCCs based on the levels of all annotated metabolites (not just the individually significant ones) and had more than one metabolite identified as a “hit” within the pathway. In order to find only the pathways enriched for docetaxel differences and not between the initial state and these treatments, only the 24 and 48 hour time points were included in this analysis. Table 2 shows the pathways that were enriched. Many of the pathways affected by docetaxel are amino acid and carbohydrate metabolism pathways, but pathways involved in nucleotide metabolism and cofactor and vitamin metabolism have also been altered.

**Table 2:** **Metabolic pathways significantly enriched for differences between control and docetaxel treated OCCs**

**KEGG pathway**	**Raw p**	**FDR**
Tyrosine metabolism	1.03E-03	0.030
Butanoate metabolism	1.87E-03	0.030
Phenylalanine metabolism	1.93E-03	0.030
Citrate cycle (TCA cycle)	3.35E-03	0.037
Glutathione metabolism	6.36E-03	0.037
Nicotinate and nicotinamide metabolism	6.60E-03	0.037
Thiamine metabolism	7.52E-03	0.037
Alanine, aspartate and glutamate metabolism	7.73E-03	0.037
Pyrimidine metabolism	9.01E-03	0.038
Arginine and proline metabolism	0.011	0.038
Beta-Alanine metabolism	0.012	0.038
Pantothenate and CoA biosynthesis	0.012	0.038
Aminoacyl-tRNA biosynthesis	0.013	0.038
Nitrogen metabolism	0.016	0.046
Purine metabolism	0.017	0.046

Growth curves of OCCs under docetaxel treatment (at both IC_50_ and 1.5 × IC_50_ values) further support the metabolic findings that docetaxel treatment alters cellular growth (shown in Additional file 2: Table S4 and Figure S1A). Overall, docetaxel does not increase the percentage of dead cells compared to the control, but it does drastically reduce the growth rate of OCCs over 48 hours.

### Uracil levels are drastically increased at 24 hours in docetaxel treated OCCs

Uracil, a nucleobase used for RNA production, allosteric regulation, and as a coenzyme, was commonly found as a contributor for differences between control and docetaxel treated cells during all analyses. In two-way ANOVA, uracil was highly significantly different in the group effect, time effect, and interaction effect in the three data sets (Table 1).

**Figure 2:**
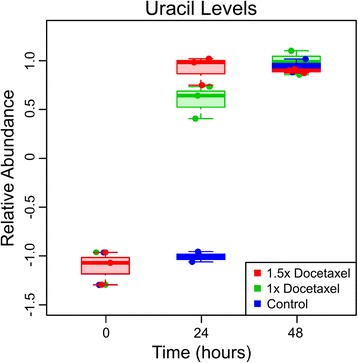
**Uracil levels plotted over time show large separation between docetaxel treated and control OCCs at 24 hours.** Time series plot of uracil levels show similar trends between the OCCs treated with the two docetaxel concentrations but a different trend for control OCCs. Box and whisker graphs depict the normalized peak area differences between the two cell types: dark lines are the median, boxes identify the middle 50% values, dashed lines show two standard deviation bounds, and circles indicate data points.

Uracil levels in docetaxel treated cells spiked at 24 hours (rising above the detection limits, while the controls were below detection limits) and then leveled off at 48 hours, at which time the uracil in the control cells reached similar levels, as shown in Figure 2. The quicker spiking of uracil levels in docetaxel treated OCCs could be the cell reacting to the stress by increasing production of uracil or the docetaxel treatment blocking pathways that utilize uracil. These docetaxel-induced changes in uracil levels are particularly noteworthy, as recent clinical studies have shown that synthetic analogs of uracil (such as 5-flurouracil or tegafur/uracil), which inhibit enzymes using uracil as a substrate, when administered in combination with docetaxel cause improved treatment results for gastric cancer, prostate cancer, and lung metastases [11-14]. Therefore, if docetaxel treatment does increase cellular dependence on uracil in the cells that are somewhat docetaxel resistant, then this would help explain why the addition of a competitive inhibitor for uracil improves patient treatment results.

### Metabolic analysis reveals small metabolic changes for OCSCs upon treatment with docetaxel for 48 hours at OCC response levels

GCxGC-MS analysis detected 167 reproducible analytes for the OCSCs, including unknown analytes and annotated analytes that were not included in human metabolic databases, with 46 unique metabolites annotated as known human metabolites. Metabolite levels for the entire data set are shown in Additional file 3: Figure S4. Univariate analysis in MetaboAnalyst revealed no significant differences (false discovery rate (FDR) corrected p value < 0.05) between the control and docetaxel treated OCSCs overall (*t* test across all time points) at the treatment level of 1.5× IC_50_ for OCCs but did detect significant differences at 24 and 48 hours individually (Additional file 2: Table S1). Further analysis using two-way ANOVA again demonstrated some changes between the control and docetaxel treated cells (Additional file 2: Table S2), with one annotated metabolite (labeled as ethanolamine) showing significant differences in the group category (Table 1).

PCA on OCSCs supported the results from the basic and time series univariate analysis. No overall separation is evident between control and docetaxel treated cells, with the main separation seen between the initial time point and the later time points (24 and 48 hours), as shown in Figure 1B, with principal component (PC) 1 fully responsible for capturing this separation. Some small and high-variability separation is evident between the control and docetaxel treated OCSCs at 48 hours; PCA on only the 48 hour time points recapitulated this separation (data not shown). Unsurprisingly, MPEA further supported PCA results. MPEA identified no pathways significantly enriched for differences between control and docetaxel treated OCSCs for 24 and 48 hours or at 24 hours only. However, MPEA found three pathways significantly enriched at 48 hours, listed in Table 3. Therefore, it appears that OCSC metabolism is slightly altered by docetaxel at 48 hours, but not before this time.

**Table 3:** **Metabolite pathways significantly enriched for differences between control and docetaxel treated OCSCs at 48 hours only**

**KEGG Pathway**	**Raw p**	**FDR**
Butanoate metabolism	2.12E-03	0.035
Tyrosine metabolism	2.31E-03	0.035
Propanoate metabolism	3.75E-03	0.042

Previous work has shown that OCSC viability does not vary greatly over 96 hours of docetaxel treatment [9]. Along with previous work, the growth curves recorded during this experiment show that OCSCs had very similar number of alive cells in the control and docetaxel treatment (shown in Additional file 2: Table S4 and Figure S1B). Overall, this lack of perturbation of metabolism until 48 hours suggests higher resistance of the OCSCs to docetaxel at a treatment level to which OCCs respond. Cancer stem cells are generally less susceptible to chemotherapeutic treatments than their cancer cell counterparts, but it is not known how susceptible their cellular metabolism is to chemotherapeutics. Here, it seems that OCSCs are able to prevent any substantial systematic change in metabolism until at least 48 hours, whether through an active role (removing docetaxel or inhibiting docetaxel uptake) or through a passive role (such as their low division rate). Either way, the OCSCs exhibit no metabolic stress during the first 24 hours of docetaxel treatment.

### Glucose deprivation, hypoxia, and an ischemia-like condition affect OCCs and OCSCs in a time-dependent manner

GCxGC-MS analysis was used to profile the metabolism of the OCCs and OCSCs over a 48 hour period of environmental perturbations including glucose deprivation, hypoxia, and the combination thereof (hereafter referred to as “ischemia” since it is meant to model ischemia, although actual ischemia itself entails even greater environmental perturbation). For OCCs, 77 intracellular analytes were reproducibly detected with 22 of these analytes annotated to unique metabolites. For OCSCs, 67 reproducibly measured intracellular analytes were detected with 21 annotated to unique metabolites. (The reason for a different number of detected analytes from the chemotherapeutic data sets is the increased number of diverse samples included in the analysis, which is challenging for the alignment step in data processing.) Metabolite 3 levels for the entire OCC and OCSC data sets are shown in Additional file 4: Figure S5 and Additional file 5: Figure S6, respectively.

Two-way ANOVA was used to analyze the data to account for changes as a function of both time and treatment. OCC and OCSC samples were analyzed with each possible environmental perturbation and the control as the “condition” factor (“All” in figures), and with separate ANOVA analyses only considering an individual perturbation and the control for the “condition” factor (identified by the name of the perturbation in figures). The number of analytes with statistically significant effects indicated by these analyses (FDR < 0.05) is shown in Additional file 2: Table S3.

**Figure 3:**
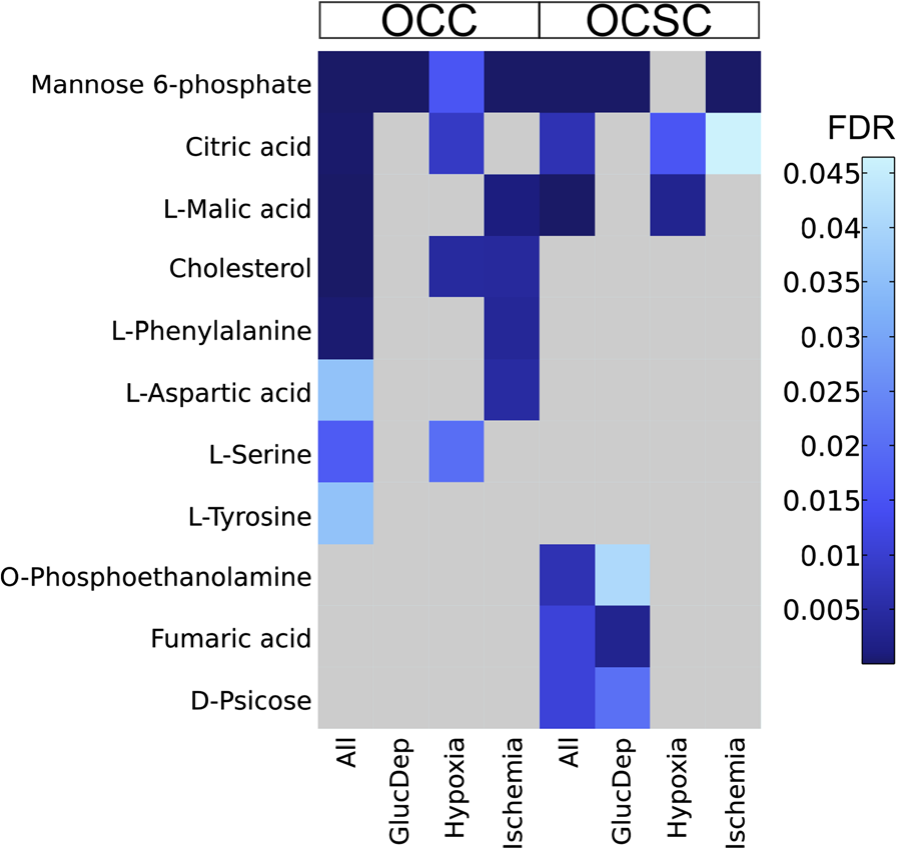
**Environmental perturbations cause different metabolite-level changes in OCCs and OCSCs.** Heatmap displays false discovery rate corrected p values for OCC and OCSC group effects for different metabolites during two-way ANOVA. Metabolites are shown in the rows, with the conditions represented in the columns. The darker the blue, the more statistically significantly different the metabolite differences are between the stated condition and the control. The three metabolites at the top represent a core, conserved set of metabolites with overall significance in both cell types.

Individual annotated metabolites found to have statistically significant group effects for all conditions or for any individual condition are shown in Figure 3 for OCCs and OCSCs. Three metabolites had significant group effects for both OCCs and OCSCs when including all conditions, representing a core of conserved metabolites with a major role in responding to one or more of these environmental stressors. One metabolite identified as significantly different in OCSCs, phosphoethanolamine, is a substrate for many cell membrane phospholipids that has recently been shown to induce both cell cycle arrest and apoptosis in cancer cells [15,16]. Here, intracellular phosphoethanolamine levels for OCSCs (and OCCs even though it is not a significant effect) stay fairly consistent over 48 hours for the control cells, but for the metabolic perturbations, the levels steadily increase over 48 hours. Because the phosphoethanolamine levels increase slightly but consistently for all metabolic perturbations, the reaction seems to be a generalized metabolic stress response. This reaction could be indicative of increased phospholipid membrane turnover or an apoptotic response to the increasing stress levels. Thus far, the apoptotic effects of phosphoethanolamine have only been studied in a controlled dose manner; [15,16] it would be interesting to determine if the cells themselves use phosphoethanolamine as an apoptotic inducer.

Other types of stress-responsive analytes are also evident in this analysis. Tyrosine would not have been detectable as having significant group effects for OCCs without the combination of all of the metabolic perturbations studied herein. This could be interpreted as a “weak core response”: not like the strong, individually significant core response observed for mannose-6-phosphate across all perturbations for OCCs, but nonetheless consistent in its small effect across all perturbations so as to yield an overall significant effect. Also of note is that there is only one significantly affected metabolite in OCCs in response to glucose deprivation, which is mannose-6-phosphate, a molecule affected in almost all conditions. This suggests a smaller-scale response of OCCs to glucose deprivation, a potentially surprising observation based on what is known about cancer metabolism and the Warburg effect.

### PCA shows separation between environmental perturbations for both OCCs and OCSCs

**Figure 4:**
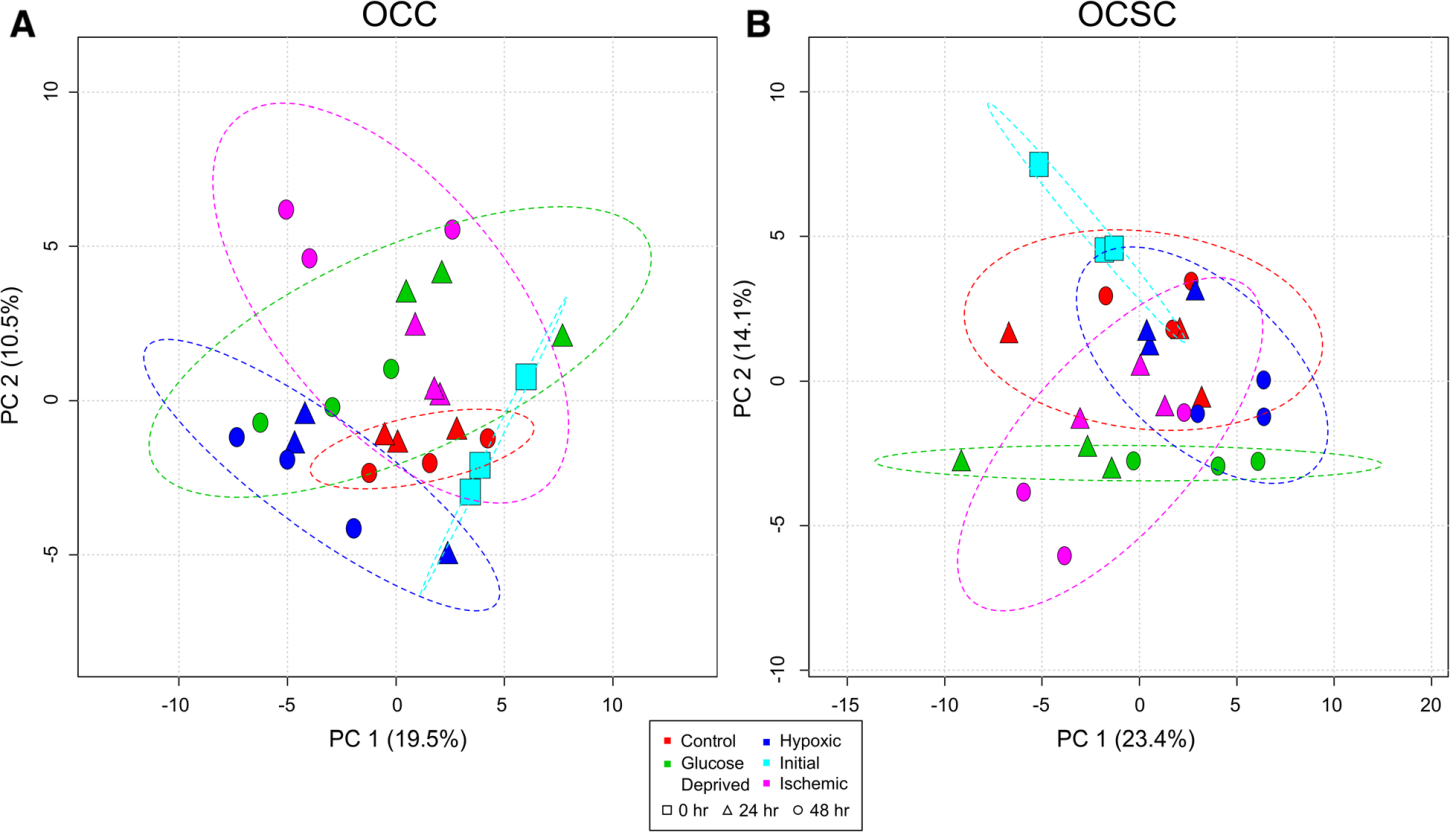
**PCA shows differences between environmental perturbations at late time points for OCCs and OCSCs.** PCA shows separation between conditions and time points at 24 and 48 hours. In both cell types, PC1 plays a large role in separating time points, and PC2 captures variation between the conditions. Dotted ovals represent 95% confidence intervals of the membership of each sample class. **A)** Control samples show little temporal variation in OCCs. Effects of ischemia are not additive based on the effects of glucose deprivation and hypoxia individually at 48 hours. **B)** OCSC control samples display much greater temporal variation compared to OCCs. There is no metabolic distinction between control and hypoxic cells, and 48-hour ischemia samples again show non-additive effects compared to glucose-deprived and hypoxic conditions.

To further characterize the effects of the perturbations on OCC and OCSC metabolism, PCA was performed on each cell type individually for all perturbations together. Plotting all conditions across all time points showed differences between the different conditions for both cell types, but only at later time points (data not shown). This suggests that the effects of these perturbations are not extremely fast, even though they are each tied closely to cellular metabolism; the impacts of these perturbations are best observed accumulated over days. For clarity and to facilitate interpretation, time points at 2, 4, and 8 hours were removed from further visualizations and analyses, and PCA was performed for all conditions at 0, 24 and 48 hours, as seen in Figure 4.

For OCCs, shown in Figure 4A, PC1 plays a large role in separating the different time points and PC2 plays a large role in separating the treatment conditions. The control and initial samples are more similar to each other than the other conditions based on their close clustering, showing that the applied perturbations induce significant changes in metabolism. The effects of ischemia, even though it is a combination of the glucose deprived and hypoxic conditions, are not additive based on the effects of glucose deprivation and hypoxia individually; instead, the 48 hour time points exhibit much greater changes for ischemia than for either of the two individual treatments.

PCA of OCSC samples again shows differences between conditions during the later time points, as seen in Figure 4B. PC1 captures significant time variance, and PC2 captures significant separation between the treatments. Similar to the OCCs, glucose deprivation shifted the OCSCs’ metabolic profile away from the control, and ischemia showed a nonadditive extra effect on metabolism at 48 hours. Unlike OCCs, though, the control samples display substantial changes and variability between time points; this is to be expected based on previous work indicating temporal changes in metabolism of OCSCs [10]. In addition, the hypoxic samples completely overlap the control, showing that hypoxia did not substantially alter the metabolic profile of the OCSCs from its normal state as it did to OCCs. Hypoxic conditions have been shown to support stemness within cancer stem cells *in vitro* and cancer stem cells have been located in hypoxic niches within the tumor [17,18]. Therefore, it is possible that OCSCs have adapted to hypoxic environments to the point where hypoxia no longer puts more stress on their metabolism compared to growth under normal oxygen concentrations.

Growth curves for OCCs and OCSCs during the environmental perturbations further support the general findings from PCA (shown in Additional file 2: Table S5 and Figure S2). As stated above, OCSCs showed much greater variablity in their growth than the OCCs overall. For the glucose deprived conditions, the number of alive OCCs stayed fairly constant over the entire 48 hour period while the number of alive OCSCs spiked at 8 hours above the control OCSCs and then dropped off over the rest of the 48 hour period, ending with a smaller number of alive cells than the control. This is a unique response of OCSCs to glucose deprivation that is not observed in OCCs. For hypoxia, OCC growth increases over the first 24 hours and then remains constant for the next 24 hours. The OCSC growth rate slows and some cells die upon being subjected to hypoxia over the first 24 hours before leveling out for the remaing time period. But according to the metabolite data, the alive cells over the experiment respond minimally to the hypoxia. Therefore, it seems that there is a subset of OCSCs that are indeed impervious to hypoxia and it is this subset that was captured in this experiment. Both cells show deviation from the control under ischemia-like conditions, with a gradual decrease in the number of alive cells.

### MPEA further supports that OCCs and OCSCs respond to hypoxia and glucose deprivation differently

**Figure 5:**
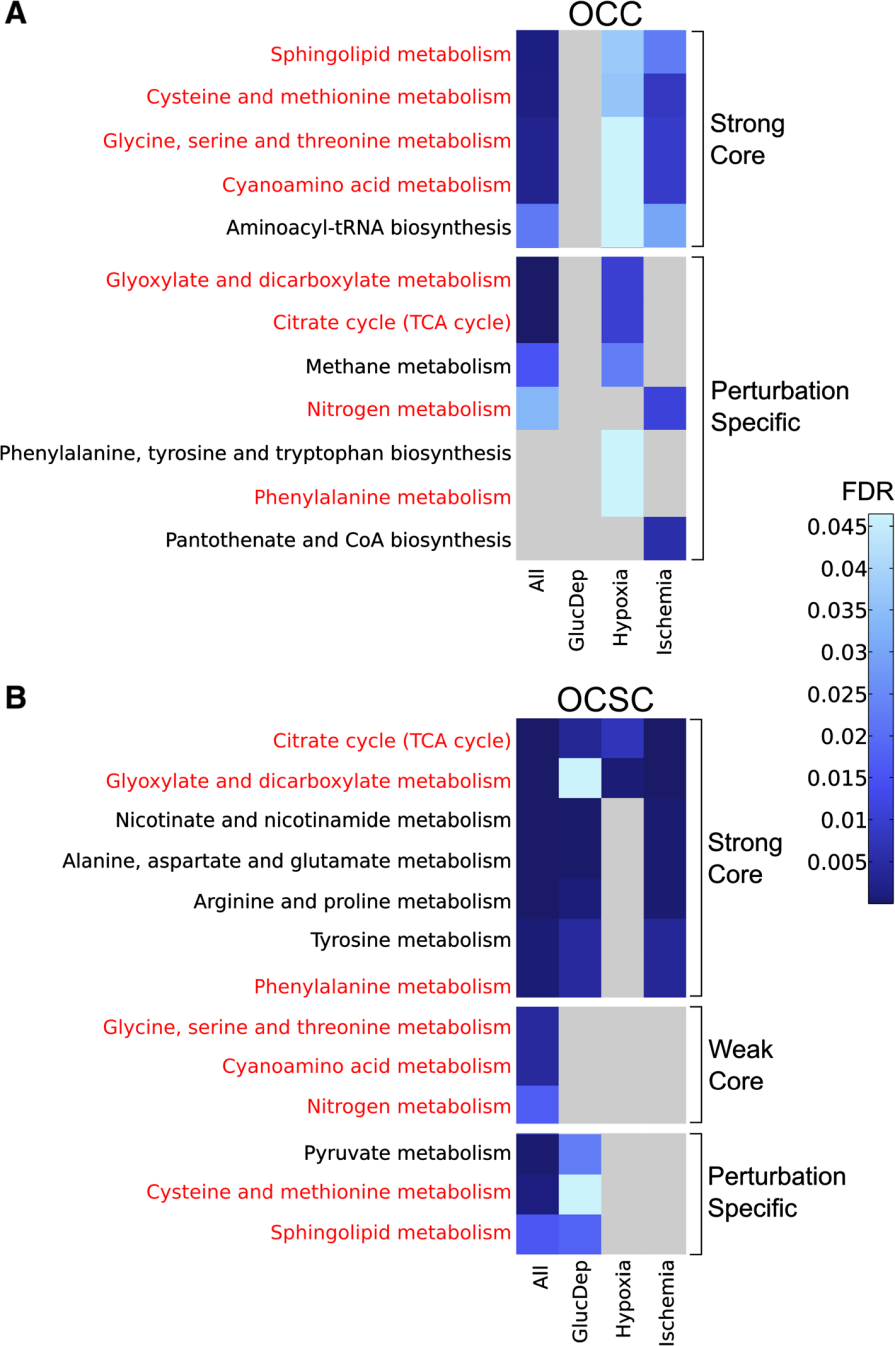
**MPEA results demonstrate trends in enriched pathways for OCCs, OCSCs, and both cell types.** Heatmap displays false discovery rate corrected p values for metabolite pathway enrichment analysis results for **(A)** OCCs and **(B)** OCSCs. KEGG pathways are shown in the rows with the conditions represented in the columns. Strong core response pathways are those with significant enrichment across all conditions and in multiple individual conditions; weak core response pathways are those enriched only across all conditions; perturbation specific pathways are those that are only enriched for a unique individual condition (which may or may not drive overall significance). The darker the blue, the more statistically significantly enriched the pathway is for metabolic differences. Pathways identified in both cell types are highlighted in red. Grey boxes represent pathways with FDR > 0.05. All: glucose deprived vs hypoxia vs ischemia vs control; GlucDep: glucose deprived vs control; Hypoxia: hypoxia vs control; Ischemia: ischemia vs control.

Using MPEA in MetaboAnalyst, 12 KEGG pathways for OCCs and 13 KEGG pathways for OCSCs were identified as having two or more metabolite “hits” and being significantly enriched (FDR < 0.05) in differences between the normal, glucose deprived, hypoxic, and ischemic conditions across all time points. Figure 5 breaks the pathway responses into three categories: “strong core” responses in either cell type are significant for multiple individual conditions as well as all individual conditions together. “Weak core” responses are significant only for all conditions together, but never for any individual condition, suggesting the combination of many small, individually insignificant effects to reflect a significant core response. The remaining class of pathways is “perturbation-specific” responses: they are significant for only one perturbation in a cell type, which may or may not drive overall significance for all conditions. Within these subtypes, we can then further identify which of these responses are conserved between cell types, and which are unique to cell types.

Overall, 8 of the statistically significantly enriched pathways overlap between OCCs and OCSCs (highlighted in red in Figure 5), indicating that many of the changes caused by the metabolic perturbations are similar between the two cells. However, 4 pathways were exclusively enriched in the OCCs and 5 pathways were exclusive for OCSCs. Detailed investigation of why these pathways were only altered in one cell type during all the perturbations may help to further explain the differences in metabolism between OCCs and OCSCs. Moreover, the specific changes in the pathways that overlap between the two cell types vary considerably; pathways that form part of the strong core in one cell type may be only perturbation-specific in another cell type, suggesting different usage of the same pathways in response to different environmental stresses.

In OCCs, glucose deprivation resulted in no significantly enriched pathways, consistent with previous analysis indicating little response of OCCs to glucose deprivation. In contrast, glucose deprivation resulted in 10 significantly enriched pathways in OCSCs that are widespread throughout metabolism, including amino acid metabolism, carbohydrate metabolism, and lipid metabolism. The differences in lipid metabolism (sphingolipid metabolism) are largely driven by differences in phosphoethanolamine levels between the control and glucose deprived cells, discussed previously. Overall, these findings suggest that OCSCs might be more dependent on glucose than OCCs, since glucose deprivation has a much larger effect on OCSC metabolism than OCC metabolism (as measured both on an individual-metabolite level and on a pathway level). This is particularly surprising given the substantially lower proliferation rate of OCSCs and the known significant glycolytic flux of the Warburg effect in bulk cancer cells.

Under hypoxic conditions, 10 pathways were significantly enriched for metabolic differences for OCCs. Out of these 10, there are four amino acid metabolism pathways enriched for statistically significant differences along with aminoacyl-tRNA biosynthesis, which prepares for translation. In all of these pathways, the amino acids driving the differences have lower levels in hypoxia than the control or are close to even. Therefore, hypoxic conditions seem to be causing significant overall changes in amino acid metabolism, whether via decreased production or increased consumption. The only metabolites with higher levels in hypoxia are citric acid and malic acid, both of which play a major role in the TCA cycle. For OCSCs, in contrast, hypoxia only resulted in two pathways being significantly enriched (glyoxylate and dicarboxylate metabolism and the TCA cycle), thus reinforcing the idea that this OCSC subset have fairly completely adapted to a hypoxic environment.

Ischemia caused 7 pathways to be significantly enriched for metabolic differences for the OCCs, two of which are perturbation-specific responses. For OCSCs, 7 pathways were statistically significantly enriched. None of these pathways are in common between the two cell types, showing that the ischemia response is very cell type-specific. Interestingly, for both cells there are some metabolic pathways that are enriched for differences under glucose deprived or hypoxic conditions that are not seen under ischemic conditions, which further reaffirms the previous observation that the effect ischemia has on the cells is not an additive effect of glucose deprivation and hypoxia.

We note that the differences between OCCs and OCSCs observed here extend the previously-observed metabolic differences between the two cell types. Of greatest interest in previous work was the difference between these cell types in arginine and proline metabolism. Differences in this pathway continue to be observed in environmental perturbations: for OCSCs, this pathway is part of the strong core response, with strong enrichment in response for all conditions except hypoxia (which exhibited essentially no response), whereas for OCCs, this pathway did not meet the inclusion thresholds. This continues to suggest that this pathway may play a key role in the metabolic differences between these cell types. Beyond arginine and proline metabolism, of the top ten most significantly enriched pathways observed in baseline differences between OCSCs and OCCs in previous work, seven of those were observed to be enriched in at least one cell type. This highlights the consistency and broad extent of changes between the two cell types.

### Limitations

For this work, only one isogenic cancer cell and cancer stem cell line pair was used. Therefore, the results found here only correspond to differences between these two particular ovarian cancer cell lines. It would be desirable to expand this study to other isogenic cancer cell and cancer stem cell line pairs, but, unfortunately, there are very few such cell lines, and they are not easily obtainable. As such cell line pairs become more widely available, these metabolic experiments should be expanded to additional cell lines to determine if the results shown here are characteristic of this specific isogenic pair or if they are indicative of broader isogenic (ovarian) cancer cell and cancer stem cell line differences.

Limitations also arise from the inherent differences between these two cell lines. OCSCs were grown as spheriods while OCCs were grown as an adherent culture which nessitated different quenching protocols for each cell line. The quenching protocols involved removing the media, washing the cells, and then quenching the metabolism within as short a time and causing as little stress to the cells as possible. These stitpulations rule out the possibility of trypsinizing the adherent OCCs, which would allow the cells to be quenched using the same protocol, as it would cause stress to the cells and add an unacceptable amount of time to the quenching protocol. Therefore, using two quenching methods was decided to be the best possible solution to this issue. Another issue that arises between the two cell lines is that they are grown in different medium. OCSCs require a different media base and growth factors in order to remain in their stem-like state. To compensate for possible medium effects, OCCs were grown in the stem cell medium and compared to OCCs grown in their normal media (R10). Any analytes found to differ between the OCCs grown in stem cell media and R10 media were removed from all the data sets used during these analyses (as detailed in the Methods section).

Another limitation of this study is the constraint of biological interpretation due to metabolite identification. Over half of the analytes that are retained in the final data set are labeled as unknown analytes, due to low match scores during our conservative metabolite identification step during processing. Additionally, the database used for metabolite pathway enrichment analysis does not include all of the metabolites identified within our data set. Therefore, there may be additional changes, especially in the metabolite pathway enrichment analysis, that are not currently detected because of lack of metabolite identification. Greater efforts must be made toward increasing the number of metabolites available within these databases for a complete understanding of the changes detected in these experiments. Finally, even conservative annotation score thresholds can yield false identifications on occasion. In addition, we note that the alignment step during data processing of the complex GCxGC-MS data is a difficult step that tends to affect the number of analytes reproducibly detected as more (and diverse) samples are added to the analysis.

Finally, our chemotherapeutic perturbation was only applied under standard cell culture conditions. As previously observed in the case of metformin [5], changes in the environment can cause changes in chemotherapeutic effects and efficacy; extended to the analyses in this work, this implies that the chemotherapeutic effects we observed may not be the same as what would be observed in an *in vivo* environment. To further characterize the metabolic changes caused by chemotherapeutics in actual tumors, future work should either include *in vivo* experiments or should involve *in vitro* application of chemotherapeutic and environmental perturbations (like those in this work) at the same time to capture possible interactions between these factors.

## Conclusions

In this study, OCCs and OCSCs from our model system were for the first time shown to have different metabolic responses to biologically-based perturbations applied *in vitro* to mimic *in vivo* tumor conditions. Docetaxel treatment had little effect on the metabolism of OCSCs, showing that these cells are chemo-resistant even on a metabolic level. Docetaxel had a substantial effect on OCCs, especially in amino acid metabolism and carbohydrate metabolism. Docetaxel also caused increased levels of uracil compared to the control, which may help explain why treatment with competitive inhibitors of uracil in conjunction with docetaxel improves tumor treatment. OCCs and OCSCs also reacted differently to glucose deprivation, hypoxia, and ischemia perturbations. Glucose deprivation alone did not have a large effect on OCC metabolism, but did perturb many pathways in the OCSCs, a surprising result based on the relative proliferation rates of the cells and the known high glycolytic flux associated with the Warburg effect and cancerous proliferation. Hypoxia had the reverse effect, changing the metabolism of OCCs more than OCSCs, likely indicative of the ability of hypoxic conditions to support cancer stem cell stemness *in vivo*. Ischemia affected the metabolism in seven pathways for each cell type with none of them overlapping between cell types, suggesting that OCCs and OCSCs respond to this stress more uniquely rather than similarly. However, the ischemia response in both cells is not simply an additive response of the glucose deprivation and hypoxia conditions, especially since one of those conditions yielded essentially no response in each cell type. Both pathway-level and metabolite-level analyses helped to identify core metabolic responses to multiple perturbations common across both cell types, with three metabolites identified as a conserved core response module, responding to multiple environmental perturbations in each cell type. Overall, these metabolic differences seen during chemotherapeutic and environmental perturbations *in vitro* help to provide much-needed detail to characterize the inherent differences in metabolism between OCCs and OCSCs; this information could potentially be used in the development of targeted treatments against OCSCs or cancer metabolism in general.

## Methods

### Cell culture

The OVCAR-3 cell line was obtained from the Developmental Therapeutic Program (DTP) of the National Cancer Institute (NCI). The OVCAR-3 ovarian cancer cells (OCCs) were cultured in R10 medium: RPMI-1640 (Cellgro, Mediatech Inc., Manassas, VA) supplemented with 10% fetal bovine serum (FBS, Invitrogen, Grand Island, NY) and 1% antibiotic-antimycotic solution (Cellgro, Mediatech Inc., Manassas, VA). Authenticity of the OVCAR-3 cell line was confirmed using short tandem repeat profiling performed by IDEXX RADIL (Columbia, MO) in October 2013. Cells were grown until confluence and subcultured at a ratio of 1:4.

Ovarian cancer stem cells (OCSCs) previously derived from a side population of OVCAR-3 were cultured as previously described [9]. Briefly, the OCSCs were cultured in ultra-low attachment petri dishes (Corning Incorporated, Corning, NY) in stem cell medium: DMEM/F12 (1:1) (Cellgro, Mediatech Inc., Manassas, VA) supplemented with 0.4% bovine serum albumin (BSA, Sigma-Aldrich, St. Louis, MO), 20 ng/mL epidermal growth factor (EGF, Invitrogen, Grand Island, NY), 10 ng/mL basic fibroblast growth factor (bFGF, Sigma-Aldrich, St. Louis, MO), 5 μg/mL insulin (Sigma-Aldrich, St. Louis, MO), and 1% antibiotic-antimycotic solution (Cellgro, Mediatech Inc., Manassas, VA). The spheroids were dissociated and reseeded at a density of 10^5^ cells/mL each week.

### Perturbation experiments

There was one chemotherapeutic and three environmental perturbations (glucose deprived, hypoxia, and ischemia). According to the American Cancer Society, a common first line treatment for ovarian cancer includes a taxane compound, such as docetaxel, which was used as the chemotherapeutic for this study. Docetaxel disrupts cellular division through suppression of microtubule dynamics in the cells, eventually leading to apoptosis [19]; we expected that this interaction would have a substantial effect on metabolism. For OCCs, two different concentrations of docetaxel dissolved in dimethylsulfoxide (DMSO) were given to the cells, the IC_50_ value (10 nM) and 50% above the IC50 value (1.5× IC_50_) (15 nM). IC_50_ values for OCCs were reported in previous work [9]. For the OCSCs, only the higher concentration of docetaxel (1.5× IC_50_ of the OCCs) was given to the cells since the higher concentration would be more likely to have an impact on the OCSCs. Solutions of docetaxel dissolved in DMSO at 100 μM and 150 μM were used to obtain the desired final required concentrations for IC_50_ and 1.5× IC_50_. An equivalent amount of DMSO was added to control media to account for effects of DMSO.

For glucose-deprived conditions, RPMI-1640 (Cellgro, Mediatech Inc., Manassas, VA) and DMEM-F12 (US Biological, Massachusetts, MA) without glucose were obtained and used to make glucose-free R10 and stem cell media as described above. For hypoxic conditions, cells were placed in a hypoxic chamber with 2% oxygen at the beginning of the experiment. Ischemic conditions were a combination of glucose deprived and hypoxic conditions.

Immediately before applying the environmental perturbations, OCCs were passaged and seeded in 6-well plates (Greiner Bio-One, Monroe, NC) with a well surface area of 6.9 cm^2^ at a density of 3×10^5^ cells/well in 2 mL of R10 medium and incubated for 24 hours to allow the cells to attach and recover. The medium was then removed, wells were washed once with phosphate buffered saline (PBS), and then 2 mL of fresh experimental medium (prepared as described above) was applied to begin the experiment. OCSCs were dissociated and seeded into ultra-low attachment 6-well plates (Corning Incorporated, Corning, NY) containing 2 mL of fresh experimental stem cell medium (prepared as described above) at a density of 3×10^5^ cells/well with a well surface area of 6.9 cm^2^. Both the OCC and OCSC experiments were performed in biological triplicate.

### Sampling protocols

For the chemotherapeutic perturbation, samples were taken at 0 minutes, 24 hours, and 48 hours. For the hypoxic and glucose deprived perturbation, samples were taken at 0 minutes, 2 hours, 4 hours, 8 hours, 24 hours, and 48 hours. The additional short-term time points were taken since the direct metabolic nature of the perturbations might cause a fairly rapid metabolic response. For OCCs, medium was removed and cells were quickly washed with 1 mL PBS at 37°C, which was aspirated off, and then 700 μL of 80:20 methanol/water solution at −80°C was added immediately. The plate was then incubated at −80°C for 15 minutes. After incubation, remaining cellular debris were harvested using a cell scraper (BD Falcon, San Jose, CA) for intracellular analysis. For OCSCs, the media-cell mixture was transferred to a filter cup (Microcheck II beverage monitor, Pall, Port Washington, NY) with a pre-wetted membrane (0.45 μm pore Express PLUS Polyethersulfone membrane, Millipore, Billerica, MA) and the medium was filtered from the cells. The cells were then quickly washed with 4 mL PBS at 37°C. The filter was then removed and placed upside down in a petri dish containing 1.5 mL of 80:20 methanol/water solution at −80°C. The samples were then incubated at −80°C for 15 minutes. After 15 minutes, the petri dish was removed and the filter was flipped over and washed using the 1.5 mL 80:20 methanol/water solution to remove any debris still caught in the filter. An extraction blank was made for the OCSCs following the same procedure above but only 4 mL PBS was washed through the filter.

For both cell types, the intracellular solution was then transferred to a microcentrifuge tube in a cold ethanol bath and centrifuged at 5,000 *g* for 5 minutes at −4°C. The supernatant was retained, and the pellet was subsequently re-extracted twice in 100 μL of the cold 80:20 methanol/water solution, with all supernatants being pooled [20]. Intracellular samples were stored at −80°C until analysis.

### Growth media experiment

To control for the differences in media between the two cell types, a secondary experiment was performed where OCCs were grown in parallel in R10 media and stem cell media for 48 hours. Intracellular samples were taken at 0, 24, and 48 hours in the same manner as described above for the OCCs. Cell counts were also taken (data not shown) and showed that OCCs grow slower in the OCSC medium than they do in their normal medium, mimicking the slower growth rate of OCSCs.

### GCxGC-MS analysis

Before derivatization, intracellular samples were vacuum concentrated in a CentriVap at 40°C until completely dry. Samples were aliquoted so as to be already normalized to cell count: cell density at sample time had been measured on a hemacytometer, and the volume that was vacuum concentrated was varied for each sample in order to yield a final concentration of 3×10^3^ live cell equivalents/μL after derivatization. The samples were derivatized following the protocol laid out by Fiehn, *et. al*. [21] Briefly, 2.5 μL of 40 mg/mL *O*-methylhydroxylamine hydrochloride (MP Biomedicals, LLC, Santa Ana, CA) in pyridine was added to the dried sample and shaken at 1400 rpm for 90 minutes at 30°C. 22.5 μL of *N*-methyl-*N*-(trimethylsilyl) trifluoroacetamide (MSTFA) + 1% trimethylchlorosilane (TMCS) (Thermo Scientific, Lafayette, CO) was then added to the samples which were then shaken at 1400 rpm for 30 minutes at 37°C. Samples were centrifuged at 21,100 *g* for 3 minutes and 10 μL of the supernatant was added to an autosampler vial. Samples were spiked with 0.10 μL of a retention time standard solution consisting of fatty acid methyl esters (FAMEs) and an internal standard of nonadecanoic acid methyl ester dissolved in dimethylformamide.

A LECO Pegasus 4D instrument with an Aglient 7683B autosampler, Agilent 7890A gas chromatograph and time-of-flight mass spectrometer (TOF-MS) was used to analyze the samples. The first column was an HP-5, 30 m long × 0.320 mm ID × 0.25 μm film thickness (Agilent, Santa Clara, CA), and the second was an Rtx-200, 2 m long × 0.25 mm ID × 0.25 μm film thickness (Restek, Bellefonte, PA). Specific autosampler, gas chromatography, and mass spectrometry methods can be found in Additional file 2.

### Data analysis

Sample runs were first analyzed in ChromaTOF (LECO, St. Joseph, MI) to determine baseline, peak area, and peak identification. Briefly, settings included a baseline offset of 0.5, automatic smoothing, 1^st^ dimension peak width of 24 seconds, 2^nd^ dimension peak width of 0.10 seconds, and a match of 700 required to combine peaks with a minimum signal-to-noise (S/N) of 5 for all subpeaks. Peaks were required to have a S/N of 10 and have a minimum similarity score of 800 before assigning a name. Unique mass was used for area and height calculation.

To align the samples, MetPP (http://metaopen.sourceforge.net/metpp.html) was used [22]. Sample files and a derivatization reagent blank file were uploaded from ChromaTOF. Unknowns were retained during the peak alignment process. The derivatization reagent blank file for OCCs or the extraction blank file for OCSCs was used to subtract peaks attributable only to sample preparation reagents from the corresponding cells’ sample files. On-the-fly alignment was used with quality control samples manually selected as the peak list for primary alignment. Peak alignment was performed using the default criteria.

After alignment, further processing of the data was done based on the procedure laid out by Dunn, *et. al*. [23]. Batch effects were removed from the intracellular data set using LOESS. To remove analytes that were not reproducibly detected, analytes for which more than half of the values were missing in the QC samples or for which the QC samples had a coefficient of variance larger than 0.5 were removed from the data set. Then, missing values were manually corrected using small value correction only if all the values were missing in the biological replicate. Annotated analytes were then compared to the Kyoto Encyclopedia of Genes and Genomes (KEGG) or the Human Metabolome Database (HMDB); if they were listed in KEGG or HMDB they were identified as metabolites. The metabolites were then verified by a manual confirmation of similarity between the annotated peak spectrum and the library spectrum. Manual confirmation resulted in tetrahydrofuran and pyruvaldehyde peaks being re-annotated from metabolite peaks to unknown peaks.

Finally, MetaboAnalyst (http://metaboanalyst.ca/) was used for statistical and enrichment analysis, applying both the statistical analysis and time series analysis modules [24]. For both analyses, remaining missing values were k-nearest neighbors (KNN) corrected. Data was filtered using the interquantile range method and then log-transformed using generalized logarithm transformation (base 2) and autoscaled.

For enrichment analysis, both metabolite set enrichment analysis (MSEA) and metabolite pathway enrichment analysis (MPEA) yielded similar results, so only MPEA results were considered further. The entire time series was uploaded as discrete data with compound names. Metabolites were properly matched to their HMDB codes before processing the data. Data processing followed the same steps as listed above for missing value imputation and data normalization. The *Homo sapiens* pathway library was used for analysis and an in-house metabolite reference library based on detectable metabolites for our system was uploaded. Global test was used for pathway enrichment analysis, with relative-betweeness centrality as the pathway topology analysis. Pathways with an FDR < 0.05 were considered significantly enriched.

### Removal of media and extraction effects

Differences potentially due to media effects were removed from all data sets. To specifically identify media effects, MetaboAnalyst was first used to analyze the OCC media control samples. Any analytes found to have statistically significant differences (t-test or two-way ANOVA, FDR < 0.05) between the OCCs grown in R10 and OCCs grown in stem cell media were then removed from the data sets to eliminate metabolic changes due to media differences.

### Availability of supporting data

The metabolomics data supporting the results of this work are available in the MetaboLights database under study identifier MTBLS150.

## Additional files

Additional file 1: Figure S3. Hierarchical clustering of analytes for OCC chemotherapeutic data set. Heatmap columns represent samples, labeled with sample type, time, and biological replicate. Rows represent hierarchically clustered analytes. Metabolite levels are mean-centered and unit-variance on a per-metabolite basis. Additional file 2:
**Supplemental Information.** Contains tables with the number of statistically significant analytes found during chemotherapeutic and environmental perturabtions for both OCCs and OCSCs, growth curves and tables with cell count information, and detailed description of methods used for autosampler, gas chromatograph, and mass spectrometer during sample analysis. Additional file 3: Figure S4. Hierarchical clustering of analytes for OCSC chemotherapeutic data set. Heatmap columns represent samples, labeled with sample type, time, and biological replicate. Rows represent hierarchically clustered analytes. Metabolite levels are mean-centered and unit-variance on a per-metabolite basis. Additional file 4: Figure S5. Hierarchical clustering of analytes for OCC environmental perturbation data set. Heatmap columns represent samples, labeled with sample type, time, and biological replicate. Rows represent hierarchically clustered analytes. Metabolite levels are mean-centered and unit-variance on a per-metabolite basis. Additional file 5: Figure S6. Hierarchical clustering of analytes for OCSC environmental perturbation data set. Heatmap columns represent samples, labeled with sample type, time, and biological replicate. Rows represent hierarchically clustered analytes. Metabolite levels are mean-centered and unit-variance on a per-metabolite basis.

## Abbreviations

ANOVA: Analysis of variance
CoA: Coenzyme A
DMSO: Dimethylsulfoxide
FDR: False discovery rate
GCxGC-MS: Two-dimensional gas chromatography–mass spectrometry
HMBD: Human metabolome database
KEGG: Kyoto Encyclopedia of Genes and Genomes
MPEA: Metabolite pathway enrichment analysis
PBS: Phosphate buffered saline
PC: Principal component
PCA: Principal component analysis
OCCs: Ovarian cancer cells
OCSCs: Ovarian cancer stem cells
