# Supplementary figures and images for: Distinct metabolic responses of an ovarian cancer stem cell line
Source: BMC Syst Biol. 2014 Dec 18;8:134. doi: 10.1186/s12918-014-0134-y (PMC4308021; doi:10.1186/s12918-014-0134-y)

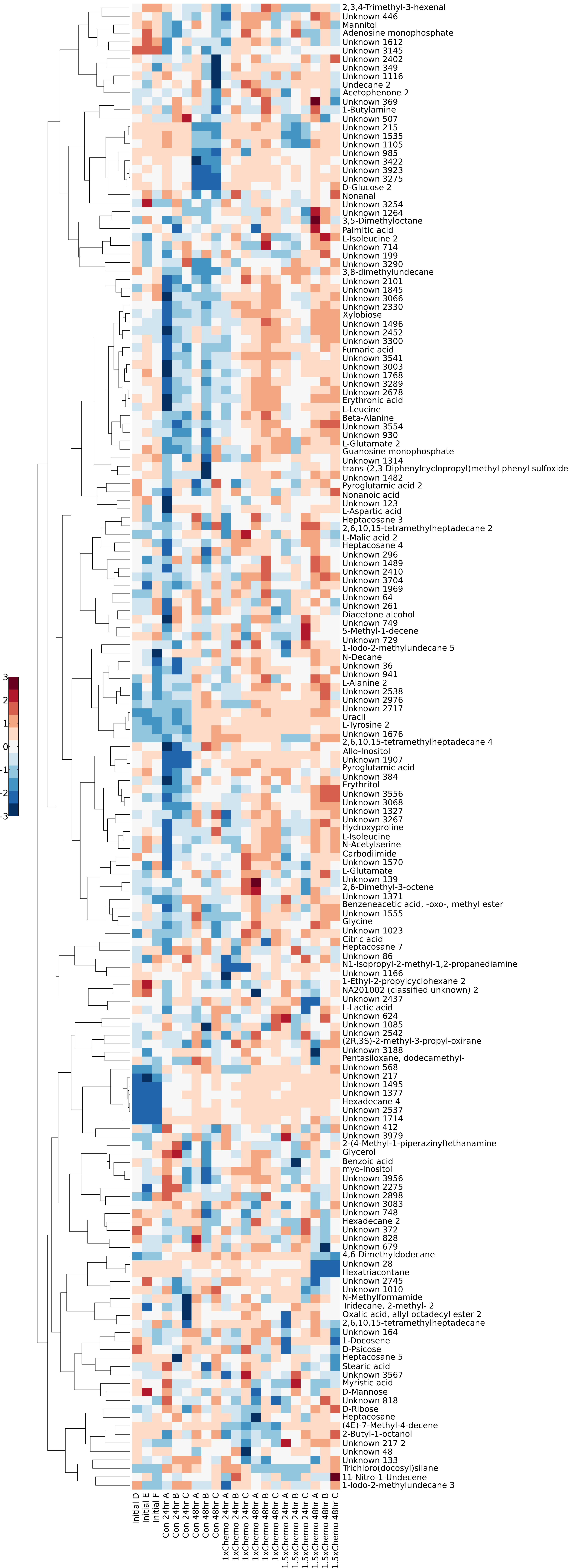

Supplement: Additional file 1: Figure S3. — Hierarchical clustering of analytes for OCC chemotherapeutic data set. Heatmap columns represent samples, labeled with sample type, time, and biological replicate. Rows represent hierarchically clustered analytes. Metabolite levels are mean-centered and unit-variance on a per-metabolite basis. [file 12918_2014_134_MOESM1_ESM.png]

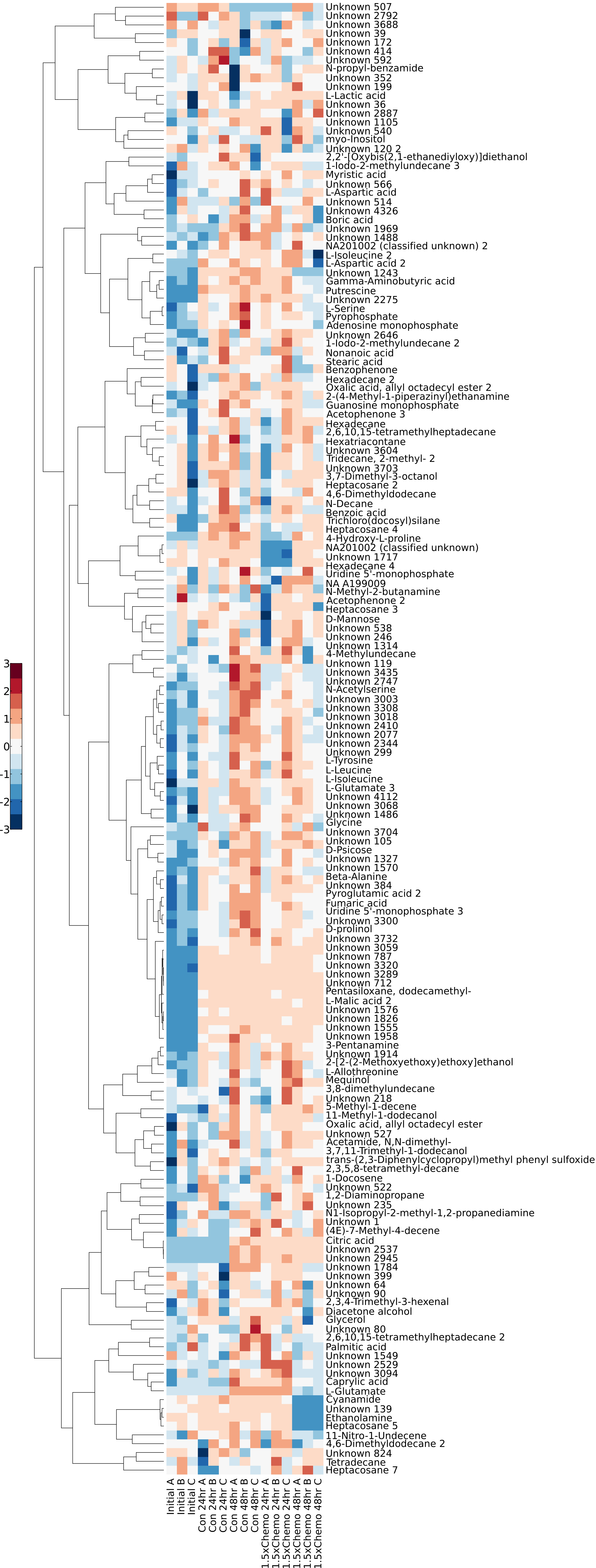

Supplement: Additional file 3: Figure S4. — Hierarchical clustering of analytes for OCSC chemotherapeutic data set. Heatmap columns represent samples, labeled with sample type, time, and biological replicate. Rows represent hierarchically clustered analytes. Metabolite levels are mean-centered and unit-variance on a per-metabolite basis. [file 12918_2014_134_MOESM3_ESM.png]

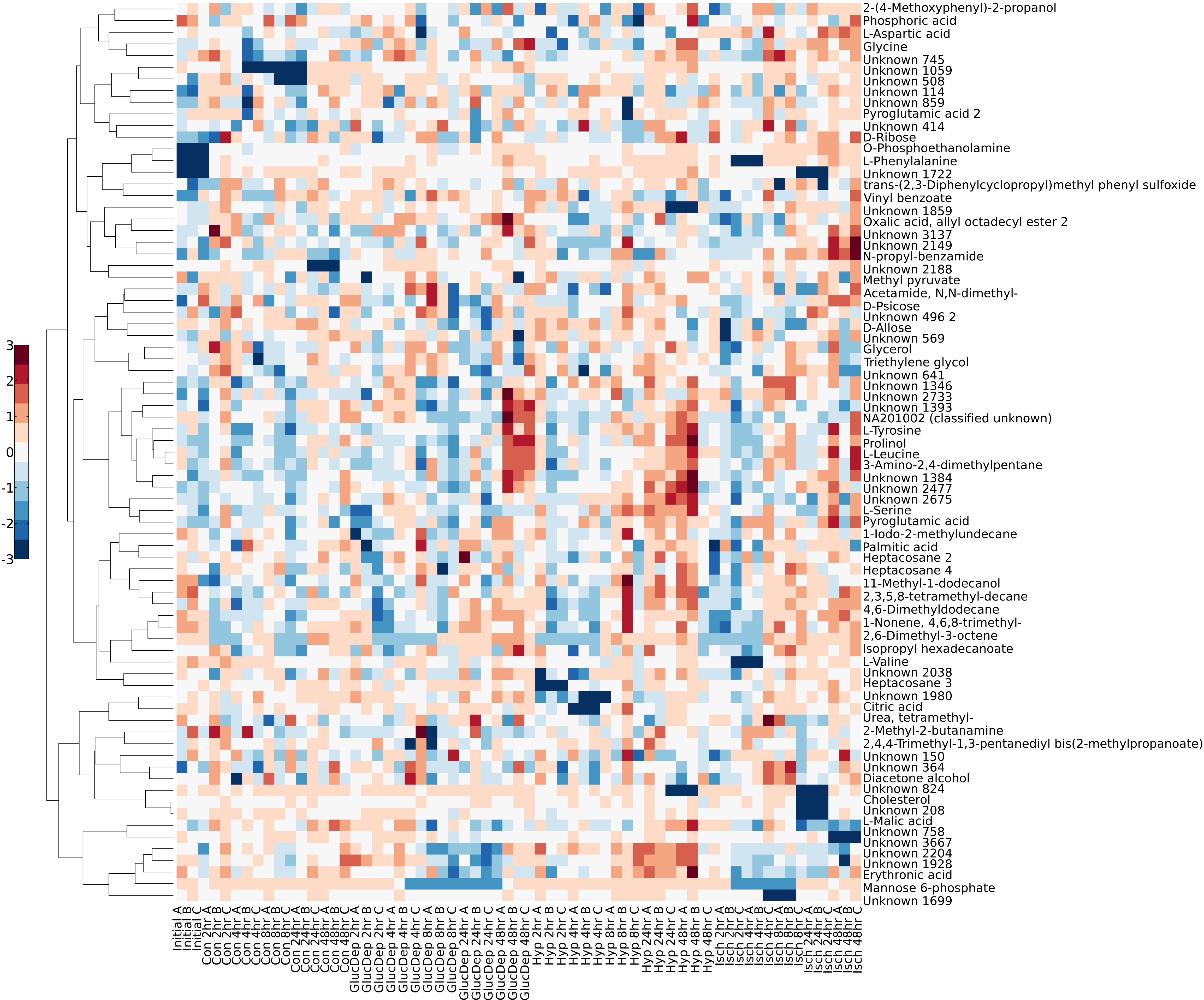

Supplement: Additional file 4: Figure S5. — Hierarchical clustering of analytes for OCC environmental perturbation data set. Heatmap columns represent samples, labeled with sample type, time, and biological replicate. Rows represent hierarchically clustered analytes. Metabolite levels are mean-centered and unit-variance on a per-metabolite basis. [file 12918_2014_134_MOESM4_ESM.png]

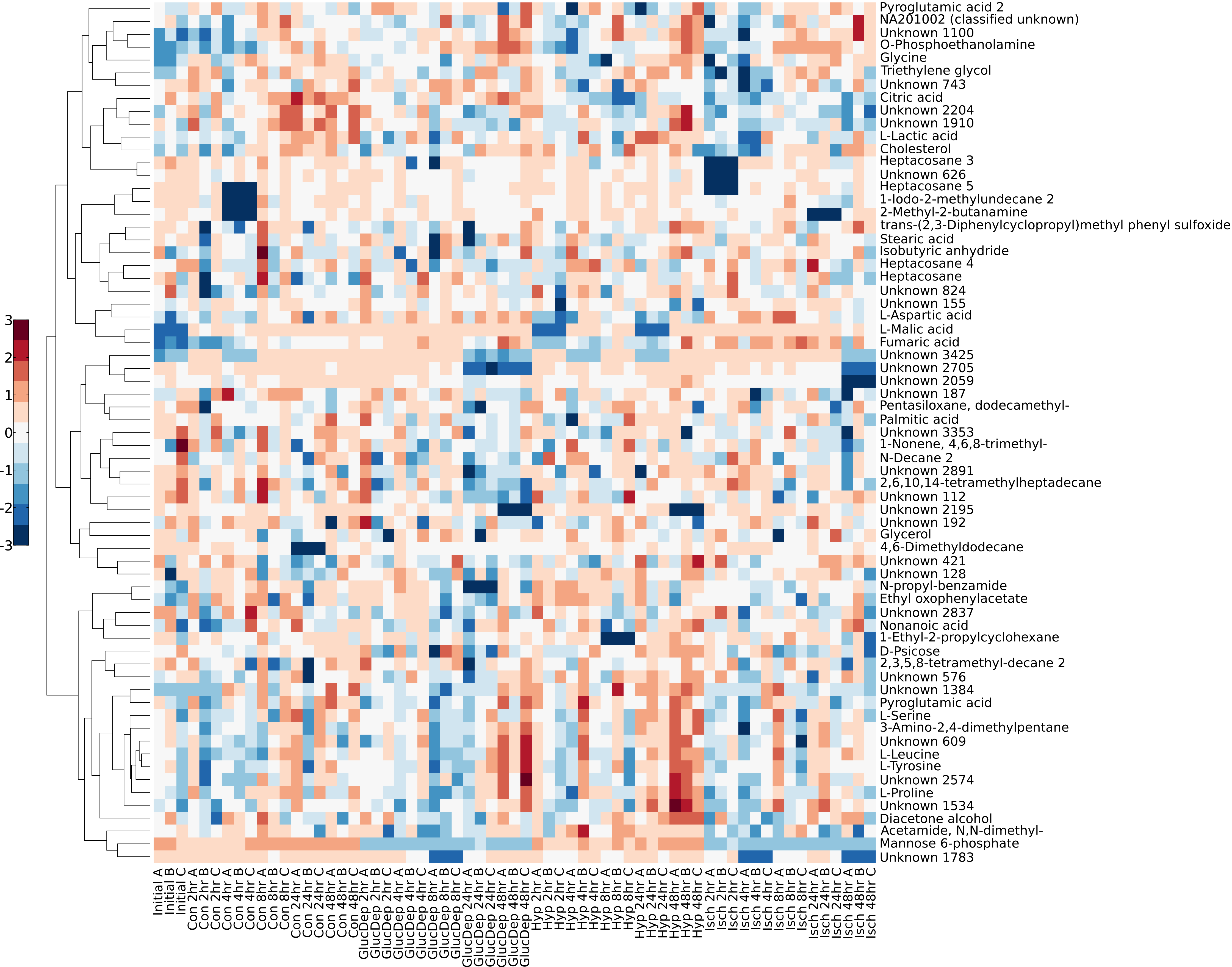

Supplement: Additional file 5: Figure S6. — Hierarchical clustering of analytes for OCSC environmental perturbation data set. Heatmap columns represent samples, labeled with sample type, time, and biological replicate. Rows represent hierarchically clustered analytes. Metabolite levels are mean-centered and unit-variance on a per-metabolite basis. [file 12918_2014_134_MOESM5_ESM.png]
