# Supplementary material for: Distinct metabolic responses of an ovarian cancer stem cell line
Source: BMC Syst Biol. 2014 Dec 18;8:134. doi: 10.1186/s12918-014-0134-y (PMC4308021; doi:10.1186/s12918-014-0134-y)
Supplement: Additional file 2: — Supplemental Information. Contains tables with the number of statistically significant analytes found during chemotherapeutic and environmental perturabtions for both OCCs and OCSCs, growth curves and tables with cell count information, and detailed description of methods used for autosampler, gas chromatograph, and mass spectrometer during sample analysis. [file 12918_2014_134_MOESM2_ESM.pdf]

Additional File 1: Supplemental Information

Number of signficantly different analytes found during chemotherapeutic treatment and environmental peturbations

Table S1: Number of statistically significant analytes found in OCC and OCSC analysis during chemotherapeutic treatment.

The number of statistically significant analytes (FDR < 0.05) found during ANOVA (Control vs IC_{50} vs 1.5x IC_{50}) or t-test analysis (Control vs IC_{50} and 1.5x IC_{50}).

		OCC			OCSC
	Time Points	Control vs IC_{50} vs 1.5 x IC_{50}	Control vs IC_{50}	Control vs 1.5 x IC_{50}	Control vs 1.5 x IC_{50}
All Analytes	All	20	9	1	0
	24 hr	16	7	11	8
	48 hr	24	5	10	8
Metabolites	All	3	3	0	0
	24 hr	2	3	2	1
	48 hr	2	2	0	3

Table S2: Number of statistically significantly different analytes in OCC and OCSC using two-way ANOVA time series analysis during chemotherapeutic treatment.

The number of statistically significant analytes (FDR < 0.05) found during time series analysis using two-way ANOVA.

		OCC			OCSC
	Category	Control vs IC_{50} vs 1.5 x IC_{50}	Control vs IC_{50}	Control vs 1.5 x IC_{50}	Control vs 1.5 x IC_{50}
All Analytes	Group	36	19	19	11
	Time	54	38	37	76
	Interactions	23	18	19	18
Metabolites	Group	7	7	5	1
	Time	9	6	4	21
	Interactions	4	4	3	4

Table S3: Number of statistically significant analytes for environmental perturbations of OCCs and OCSCs using two-way ANOVA.

The number of statistically significant analytes (FDR < 0.05) found during time series analysis using two-way ANOVA.

		All Analytes			Metabolites Only		
		Group	Time	Interactions	Group	Time	Interactions
OCC	All Conditions	22	59	32	8	14	9
	GlucDep vs Con	3	27	7	1	7	2
	Hypoxia vs Con	7	29	9	4	6	2
	Ischemia vs Con	15	27	22	5	7	7
OCSC	All Conditions	18	40	19	6	12	5
	GlucDep vs Con	7	20	12	4	7	2
	Hypoxia vs Con	5	23	9	2	6	2
	Ischemia vs Con	9	26	11	2	8	1

Growth Curves during chemotherapeutic treatment and environmental peturbations

Table S4: Growth information for OCCs and OCSCs during chemotherapeutic treatment.

Total cell count, standard error of the mean (SEM), percentage of alive cells, and percentage of dead cells for control and docetaxel treatments for OCCs and OCSCs at 0, 24, and 48 hours.

TP	Cell Count	OCC			OCSC	
		Control	IC50	1.5xIC50	Control	1.5xIC50
0	Total	4.45E+05			2.40E+05	
	SEM	3.62E+04			N/A	
	Alive %	91%			72%	
	Dead %	9%			28%	
24	Total	1.35E+06	4.78E+05	6.36E+05	2.04E+05	2.09E+05
	SEM	5.66E+04	3.04E+04	2.55E+04	N/A	N/A
	Alive %	90%	72%	87%	69%	64%
	Dead %	10%	28%	13%	31%	36%
48	Total	1.42E+06	5.85E+05	4.64E+05	1.63E+05	2.47E+05
	SEM	1.13E+04	1.16E+05	5.09E+04	N/A	2.17E+04
	Alive %	78%	63%	90%	61%	53%
	Dead %	22%	37%	10%	39%	47%

Table S5: Growth information for OCCs and OCSCs during environmental perturbations.
Total cell count, standard error of the mean (SEM), percentage of alive cells, and percentage of dead cells for control and environmental perturbations for OCCs and OCSCs at 0, 8, 24, and 48 hours.

TP	Cell Count	OCC				OCSC			
		Control	GlucDep	Hypoxia	Ischemia	Control	GlucDep	Hypoxia	Ischemia
0	Total	4.96E+05				1.29E+05			
	SEM	1.81E+05				1.56E+04			
	Alive %	96%				81%			
	Dead %	4%				19%			
8	Total	5.88E+05	6.00E+05	4.48E+05	4.68E+05	1.20E+05	2.17E+05	1.48E+05	1.39E+05
	SEM	4.81E+04	9.05E+04	9.05E+04	2.55E+04	6.55E+03	7.54E+03	8.49E+03	2.98E+04
	Alive %	90%	94%	92%	91%	77%	79%	74%	62%
	Dead %	10%	6%	8%	9%	23%	21%	26%	38%
24	Total	9.64E+05	7.96E+05	1.05E+06	7.12E+05	1.36E+05	1.87E+05	7.90E+04	1.46E+05
	SEM	5.37E+04	1.22E+05	9.33E+04	1.47E+05	1.89E+03	7.54E+03	3.48E+03	3.11E+04
	Alive %	90%	77%	84%	80%	70%	79%	49%	46%
	Dead %	10%	23%	16%	20%	30%	21%	51%	54%
48	Total	1.51E+06	5.16E+05	7.96E+05	2.36E+05	1.89E+05	1.31E+05	7.02E+04	1.64E+05
	SEM	2.01E+05	3.68E+04	1.39E+05	1.98E+04	4.24E+03	7.07E+03	1.18E+04	3.57E+04
	Alive %	91%	81%	92%	93%	75%	53%	44%	20%
	Dead %	9%	19%	8%	7%	25%	47%	56%	80%

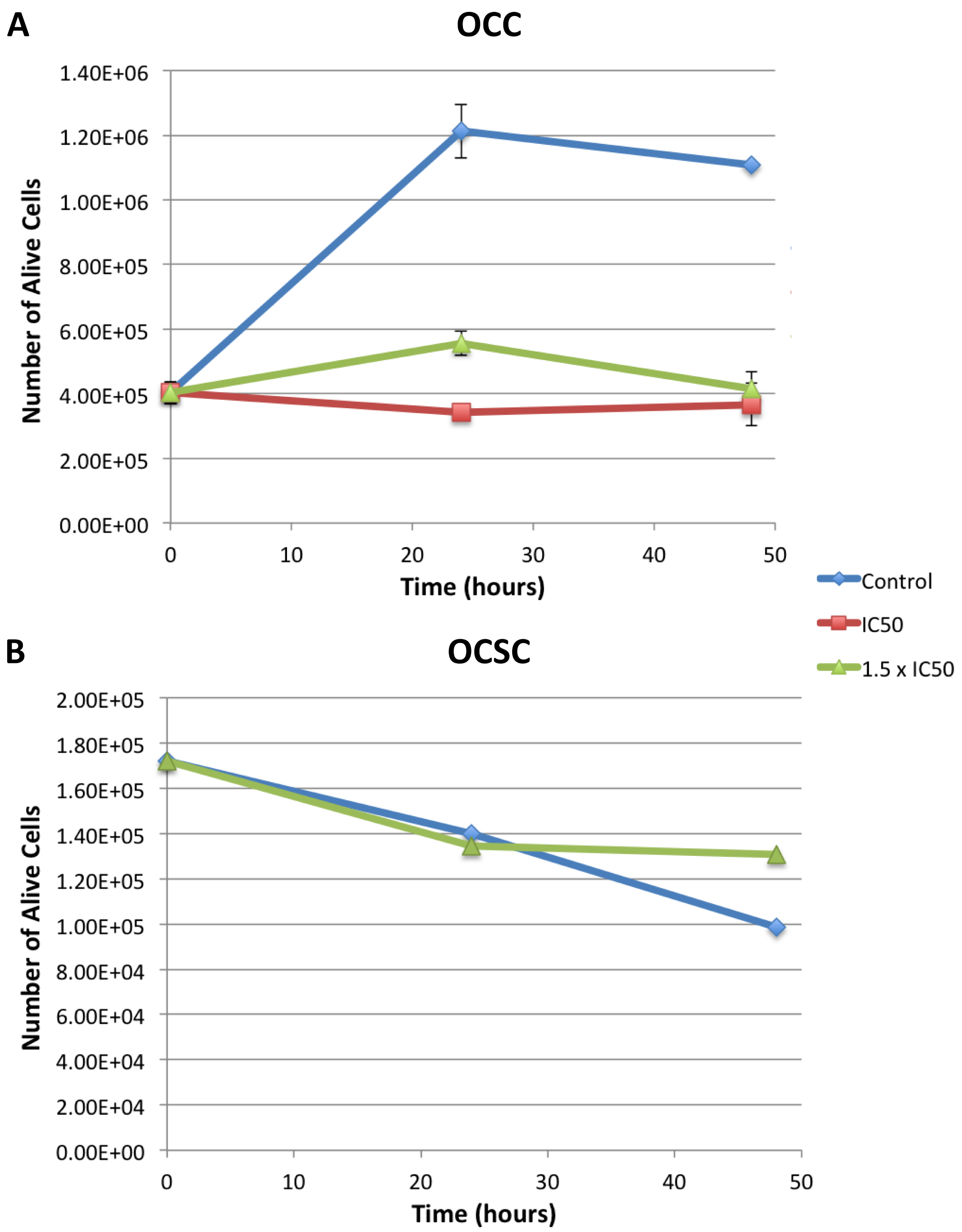

Figure S1: Growth curves during chemotherapeutic treatments for OCCs and OCSCs. Number of alive cells at 0, 24, and 48 hours for (A) OCCs and (B) OCSCs for the control and docetaxel treatment levels during the chemotherapeutic experiments.

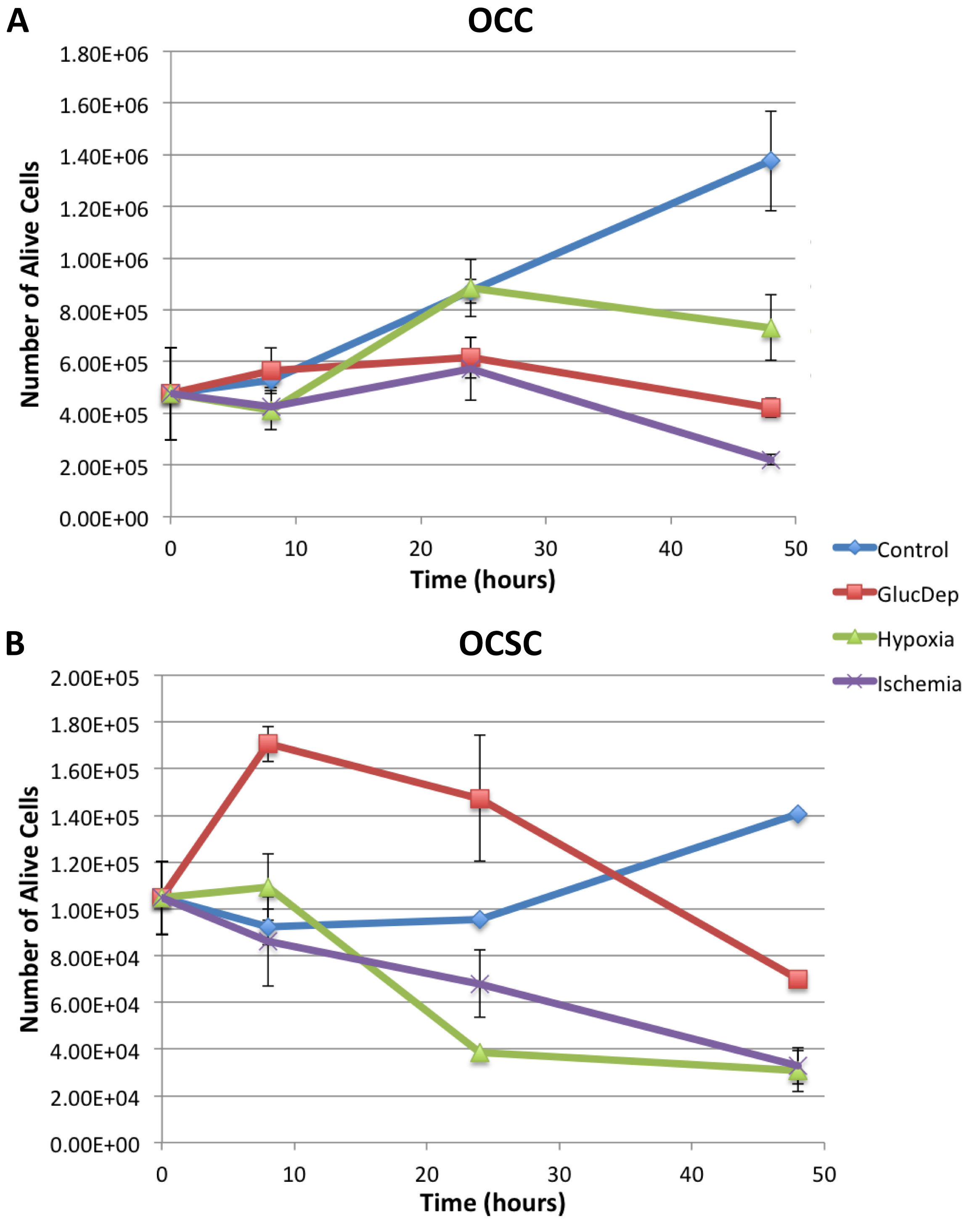

Figure S2: Growth curves during environmental perturbations for OCCs and OCSCs. Number of alive cells at 0, 24, and 48 hours for (A) OCCs and (B) OCSCs for the control and each environmental perturbation during the chemotherapeutic experiments.

GCxGC-MS Methods

AS Method

An Agilent 7683 autosampler was used. Three pre-washes with pyridine were performed before each injection. The sample was then pumped into the syringe 4 times. The syringe size was 10 μL with a sample volume of 1 μL injected into the inlet. Three post-washes with pyridine were performed after injection.

GC Method

An Agilent 7890 gas chromatograph adapted to GCxGC analysis was used. The first column was an HP-5, 30 m long x 0.320 mm ID x 0.25 μm film thickness (Agilent, Santa Clara, CA), and the second was an Rtx-200, 2 m long x 0.25 mm ID x 0.25 μm film thickness (Restek, Bellefonte, PA). The excluded masses in auto mass defect mode option was chosen. Helium was used as the carrier gas with a corrected constant flowrate of 1.00 mL/min. An inlet septum purge flow of 3mL/min was chosen. The inlet was operated in splitless mode with a purge flow of 100 mL/min set to start 30 seconds after injection, yielding a total flow of 101 mL/min. Gas saver mode was used, with a flow of 20 mL/min set to start a minute after injection.

Table S6: Main Oven Temperature Program for Intracellular and Extracellular Samples

Rate (°C/min)	Target Temp (°C)	Duration (min)
Initial	70	1
10	315	2

The main oven temperature program can be found in Table S6. The secondary oven temperature offset was 5°C and the modulator temperature offset was 20°C from the main oven. An equilibration time of 60 seconds was set for the ovens. The modulation timing is listed in Table S7. The transfer line was set to 320°C for the entire run.

Table S7: Modulation Timing

#	Start (s)	End (s)	Modulation Period (s)	Hot Pulse Time (s)	Cold Pulse Time (s)
Intracellular Samples					
1	Start	575	5.00	1.00	1.50
2	575	797	6.00	1.50	1.50
3	797	End	4.00	1.50	0.50
Extracellular Samples					
1	Start	492	4.00	0.50	1.50
2	492	747	5.00	1.00	1.50
3	747	1099	4.00	1.00	1.00
4	1099	End	4.00	1.50	0.50

MS Method

A LECO Pegasus IV D time of flight mass spectrum (TOF-MS) was used. The total MS method time was based on the GC method time. The acquisition delay was set to 240 seconds, with the filaments being turned off until then. The collection mass range was from 50 to 500 u. The acquisition rate was set to 200 spectra/second. The detector voltage was set to 100 V above the optimized voltage with the electron energy set to -70 V. The mass defect mode was set to manual with the mass defect 0 mu/ 100 u. The ion source temperature was 220°C and the run had to wait for the ion source temperatures to reach the set point before starting acquisition.
